# Associations between out-of-home care and mental health disorders within and across generations in a Swedish birth cohort

**DOI:** 10.1016/j.ssmph.2022.101115

**Published:** 2022-05-08

**Authors:** Viviane S. Straatmann, Josephine Jackisch, Lars Brännström, Ylva B. Almquist

**Affiliations:** a Department of Public Health Sciences, Stockholm University, Sweden; b Department of Social Work, Stockholm University, Sweden

**Keywords:** Intergenerational transmission, Out-of-home care, Mental disorders, Sex differences, Cross-lagged panel model

## Abstract

Previous studies have shown that mental health disorders (MHD) among parents might be an important mechanism in the intergenerational transmission of out-of-home care (OHC). The current study aimed to further study this interplay by investigating the associations between OHC and MHD within and across generations. We used prospective data from the Stockholm Birth Cohort Multigenerational Study (SBC Multigen) on 9033 cohort members (Generation 1; G1) and their 15,305 sons and daughters (Generation 2; G2). By odds ratios of generalised structural equation modelling, we investigated the intergenerational transmission of OHC and MHD, respectively, as well as the association between OHC and MHD within each generation. Second, we examined the associations between OHC and MHD across the two generations. In order to explore possible sex differences, we performed the analyses stratified by the sex of G2. The results showed an intergenerational transmission of OHC, irrespective of sex. Regarding the intergenerational transmission of MHD, it was shown for both sexes although only statistically significant among G2 males. OHC was associated with MHD within both generations; in G2, this association was stronger among the males. While we found no direct association between OHC in G1 and MHD in G2, there was a significant association between MHD in G1 and OHC in G2. The latter was more evident among G2 females than G2 males. We conclude that OHC and MHD seem to be processes intertwined both within and across generations, with some variation according to sex. Although there did not seem to be any direct influences of OHC in one generation on MHD in the next generation, there was some indication of indirect paths going via parental MHD and child OHC.

## Highlights

• Mental health disorders (MHD) (G1) was directly associated with out-of-home care (G2) among females.
• There were no direct influences of OHC in one generation on MHP in the next generation.
• There was some indication of indirect paths going via parental MHD and child OHC.

## 1 Introduction

Placing children in out-of-home care (OHC) is aimed to provide them with better developmental opportunities than in their home of birth, as these children are often exposed to adversities (e.g. maltreatment, neglect, psychosocial problems, socioeconomic disadvantages) or struggle with serious conduct problems and delinquency themselves. Nevertheless, adverse circumstances tend to be transmitted across generations. The disadvantage rooted in individual experiences may grow over time; this results in their descendants starting at a comparatively higher level of disadvantage making it difficult for them to catch up (DiPrete and Eirich, 2006). Reflective on this, a growing number of studies show that children are at greater risk of placement in OHC when their parents had a history of OHC (Jackson Foster et al., 2015; Mertz and Andersen, 2017; Straatmann et al., 2021; Wall-Wieler et al., 2018a, 2018b). In addition, mental health is an important aspect linked to the experience of being placed in OHC (Heim et al., 2008; Park et al., 2019). The mental health needs of these children tend to perpetuate after ageing out of care (Baldwin et al., 2019; Oswald et al., 2010; Hambrick et al., 2016; K ää ri ä l ä and Hiilamo, 2017), a period when parenthood is likely to occur which poses a risk for mental health disorders (MHD) and other adversities into the next generation (Fusco, 2015; Plant et al., 2018; Zhukova, 2020). In particular, the evidence regarding intergenerational transmission of MHD is strong (Goodman, 2020; Hancock et al., 2013; Maciejewski et al., 2018), although the contribution of hereditary components (Sullivan et al., 2012) and environmental factors in such association is much debated (Guintivano and Kaminsky, 2016; Park et al., 2019).

Therefore, children in OHC are a vulnerable group with great mental health needs that may require interventions and support from different institutional systems. In Sweden, regional councils have the responsibility for treating people with mental disorders (i.e. health care services)(NORDON- Sommer, 2017) while municipalities offer particular mental health support through social services. In turn, the Swedish child welfare service which is often described as family service-oriented (i.e. aiming at early support and intervention to prevent OHC placements) is regulated by national legislation, however, operated by each municipality-with substantial organisational and procedures variations due to their local degree of autonomy and sociodemographic differences (Socialstyrelsen, 2020). According to regulation, all children entering into care are entitled to have a complete health examination (physical, mental and dental health), but efforts yet need to be done in order to deliver such deserved service to these children (SOU 2021:34, 2021SOU 2021:34).

Whereas some theories (e.g. social learning theory, attachment theory, trauma-based models) (Yang et al., 2018) contribute to the understanding of the continuity of OHC and other adversities across generations (Straatmann et al., 2021), it is worth speculating in a more comprehensive way how such adversities may interplay across generations (Shonkoff & Garner, 2012). The ecobiodevelopmental theoretical framework postulates that early negative experiences can leave a lasting signature on the genetic predispositions that affect emerging brain architecture, and might lead to lifelong impairments in both physical and mental health. Moreover, it also posits that such toxic circumstances play an important causal role in the intergenerational transmission of social and health outcomes disparities (Shonkoff, 2010; Shonkoff & Garner, 2012).

That said, our recent publication investigated the role of MHD in the intergenerational transmission of OHC in a multigenerational Swedish birth cohort (Straatmann et al., 2021). We found that OHC was transmitted across two generations and that MHD among parents plays a relevant role in such transmission regardless of family social class. While we found evidence for the parents' mental health impacting their child's placement, the reverse might also be true. On the one hand, the parental’ MHD can impact the child generation's risk of placement in OHC and, on the other hand, the parental generation's OHC experiences can influence the child generation's mental health. In other words, there are reasons to expect a bidirectional association between generations. For instance, in terms of the former (MHD in the parental generation being associated with OHC in the child generation), a Swedish study using a population-based retrospective cohort identified mental health illness, substance abuse, and developmental disabilities as strong predictors of mothers having their children taken into care (Wall-Wieler et al., 2018c). In terms of the latter (OHC in the parental generation being associated with MHD in the child generation), it is possible to draw a parallel to the abundant research on child maltreatment which systematically has shown that mother's history of maltreatment is associated with children's emotional and behavioural difficulties across childhood and adolescence (Plant et al., 2018; Su et al., 2020) which might lead to later MHD in adulthood.

In addition to this, while it is well known that rates of and susceptibility to MHD differ between males and females (Riecher-R ö ssler, 2017; WHO, 2001), there are a few and not consistent findings on sex differences in OHC in terms of MHD (Baldwin et al., 2019; Cotton et al., 2020; Harpin et al., 2013; Heflinger et al., 2000; Tarren-Sweeney and Hazell, 2006). On top of that, studies investigating intergenerational associations between these two constructs (i.e. parental experiences of OHC (or maltreatment) and offspring's mental health (Plant et al., 2018; Su et al., 2020; Yoon et al., 2019) or between parents’ mental health and their children's OHC experience (Hammond et al., 2017; Wall-Wieler et al., 2018c)) have not paid much attention to sex differences. Many of these studies investigated only maternal circumstances (not paternal), in addition to the fact that the children's sex was used as a covariate, without discussing the potential implications to the results.

As previously mentioned, in our first study (Straatmann et al., 2021), we aimed to explore the role of MHD in the intergenerational association of OHC, with an explicit focus on social class disparities. However, important knowledge gaps remained unexplored. Given the richness of the data material that has been used – the Stockholm Multigenerational Birth Cohort Study (SBC Multigen) – we wish to expand the knowledge by analysing the intergenerational transmission of OHC and MHD while simultaneously exploring the potential associations between OHC and MHD across two generations, disaggregated by sex.

## 2 Methods

### 2.1 Data material

Data were drawn from the Stockholm Birth Cohort Multigenerational Study (SBC Multigen). Information about the SBC Multigen cohort profile (Profile, 2020) and the initial set-up for the current study design (Straatmann et al., 2021) have been previously published.

Among the 14,608 individuals that were defined as Generation 1 (G1), 11,338 individuals had at least one child and were thus included in the study sample. Their 24,929 children formed the current study's Generation 2 (G2). The two main types of information used in the study were ‘Out-of-home care (OHC)’ and ‘Mental health disorders (MHD’. To facilitate comparisons between the generations, we restricted the occurrence of OHC placements to ages 0–12 (teenage placements were excluded since they commonly are due to mental health disorders or behavioural difficulties in the child, which would overlap with the MHD measure), and MHD to the early-mid adulthood period, i.e. between age 20–32 (to ensure the same follow-up for both generations). Since all G1 individuals were born in 1953, we thus considered OHC and MHD that occurred between 1953-1965 and 1973–1985, respectively. For G2, we first restricted the study sample to those born between 1973 and 1985 (due to data availability). OHC and MHD reflected different time periods depending on the birth year of the child. For instance, if a G2 individual was born in 1985, we measured OHC from 1985 (age 0) to 1997 (age 12), and MHD from 2005 (age 20) to 2017 (age 32). The restrictions in birth years, age of placements (from birth to 12 years old) and age of MHD occurrence rendered a sample of 15,305 individuals in G2, which constitute the main analytical sample of this study, as well as those parents that were part of the SBC Multigen (G1; n = 9033).

### 2.2 Variables

#### 2.2.1 Out-of-home care (OHC)

Information about OHC placements in G1 was based on the decisions made by the Child Welfare Committee, as recorded in the local social registers of Stockholm municipalities. As previously mentioned, we considered placements that occurred in the period between 1953 and 1965 (ages 0–12). In G2, information on OHC placement was obtained through the National Register on Social Services for Children and Youth for the period from 1973 to 1997 (ages 0–12). Variables indicating placement in OHC were coded as ‘No occurrence of OHC’ and ‘Occurrence of OHC’ for G1 and G2, respectively.

#### 2.2.2 Mental health disorders (MHD)

The Patient Register was used to extract data about MHD in G1 and G2. Records of inpatient care (at least one overnight stay at the hospital) with a diagnosis reflecting mental and behavioural disorders (Chapter F in the International Classification of Diseases, 10th revision, subsequently called ICD 10, as well as the corresponding chapters in the 8th and 9th revisions) were included. Since we restricted the occurrence of MHD to ages 20–32 in each generation. MHD in G1 was considered between 1973 and 1985, whereas in G2, MHD registered between 1993 and 2017 were included. Substance use disorders, which might have a different aetiology/mechanism and rather reflect behavioural types of disorder use (ICD 10: F10-19), as well as diseases with early-onset and a strong genetic component (e.g. autism, attention-deficit hyperactivity disorders), were not included (ICD 10: F71–F79; F80-89; F90–F98). For both generations, MHD was operationalised as binary variables: ‘No occurrence of MHD’ and ‘Occurrence of MHD’.

#### 2.2.3 Covariates

Information about G1's childhood occupational class was obtained through the Occupational register from 1953 (reflecting the occupation of the head of the household, typically the father, at the time of the birth of the G1 member). These data were classified into two categories: ‘Middle/upper class’ (middle class + upper-middle/upper class) and ‘Working class’ (working class, skilled + working-class, unskilled + unclassified). The biological sex of G1 and G2 at birth (Males; Females), respectively, were also included. The sex of G1 was included in the analysis as a covariate whereas the sex of G2 was used to stratify the analysis.

### 2.3 Statistical analysis

First, we carried out descriptive analysis using Chi-Square tests to examine sex differences in the respective generation.

A cross-lagged panel analysis was subsequently employed to estimate the associations between OHC and MHD within and across the two generations, while adjusting for the covariates (e.g. occupational class (G1) and sex (G1)). Although we acknowledge that this kind of analysis makes a causal appeal (as expressed by the nomenclatures commonly used), we prefer to be cautious and assume that we are just evaluating statistical associations. As the key variables of interest are binary, Generalised Structural Equation Modelling (GSEM) (Stata/SE 16.1) was applied with a logit function to estimate the models. GSEM allows for the structural relationships between continuous, binary, categorical, and ordered measures to be modelled using linear, logistic, multinomial and ordinal logistic specifications, respectively (Stata, 2015).

**Fig. 1:**
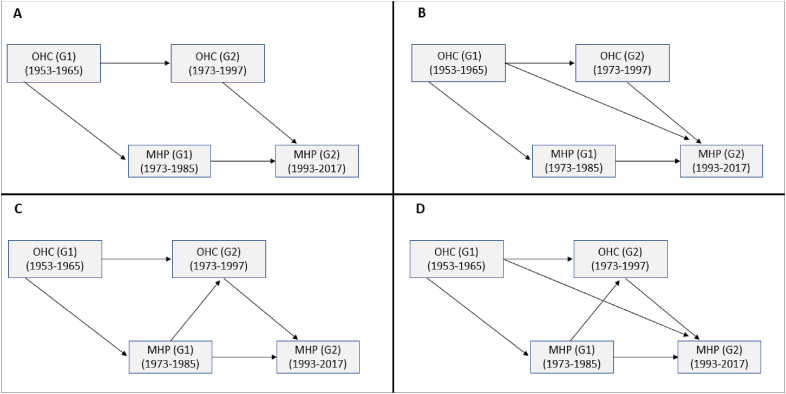
GSEM models estimate the associations between out-of-home care (OHC) and mental health disorders (MHD). Model A: Intergenerational transmission and intragenerational associations; Model B: Model A + Intergenerational association/addition of OHC (G1) predicts MHD (G2); Model C: Model A + Intergenerational association/addition of MHD (G1) predicts OHC (G2); Model D: Model A + The intergenerational associations specified in Models B and C. All models were adjusted for childhood occupational class (G1), sex (G1) – for clarity, these variables are omitted in Fig. 1.

We performed the analysis in four stages (see Fig. 1). First, we generated a model (A) to estimate the intergenerational transmission of OHC and MHD, respectively (OHC G1→ OHC G2; MHD G1→ MHD G2). This model also investigates the intragenerational associations between OHC and MHD (OHC G1→ MHDG1; OHC G2→ MHD G2). A second model (B) incorporated the association between OHC in G1 and MHD in G2. Then, a third model (C) estimated the association between MHD in G1 and OHC in G2. In the last model (D), both intergenerational associations between OHC and MHD were estimated. Covariates were included in all models. By design, we were consequently able to explore potential indirect pathways (a. OHC (G1)→MHD (G1)→OHC (G2); b. OHC (G1)→MHD (G1)→MHD (G2); c. OHC (G1)→OHC (G2)→MHD (G2)).

The ‘group function’ of GSEM was employed to retrieve model estimates separately for G2 males and G2 females. In order to verify statistical differences between sexes, overlapping CIs were analysed. The Akaike information criterion (AIC) and Bayesian information criterion (BIC) criteria were used to compare model fit between models A, B, C, and D. Models with relatively lower AIC and BIC estimates were judged as having a better fit. In this study, GSEM was estimated using the Bernoulli family with a logit link. As a result, exponentiated coefficients (Odds Ratios/OR) are reported. All analyses were performed using Stata/SE16.1.

## 3 Results

**Fig. 2:**
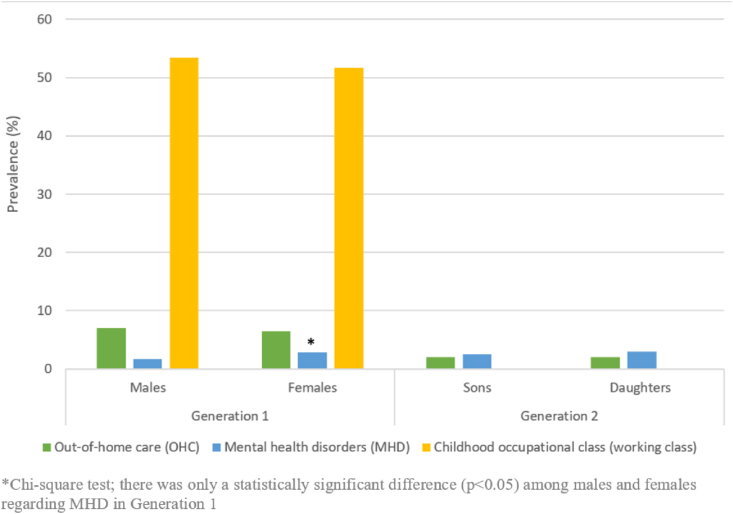
Characteristics of generations 1 and 2 by sex: out-of-home care (OHC), mental health disorders (MHD) and childhood occupational class (i.e. working class).

Fig. 2 shows that nearly 7% and 2% of individuals from G1 and G2 had experiences of being placed in OHC, respectively (without statistically significant differences between the sexes). Regarding MHD, only in G1, there was a statistically significant difference between the sexes; 1.7% of males and 2.8% of females had MHD (*p<0.05*). In G2, nearly 3% of males and females had MHD (also shown in table format at SM 1).

Table 1 shows the fit statistics for the models; each subsequent model (A-D) involves the inclusion of an additional pathway. Model C had the best fit (lower BIC for the full sample analysis, and lower AIC and BIC for the analysis grouped by sex (G2)) in comparison to models A, B, and D. Model C includes the pathway from MHD (G1) to OHC (G2) but not the pathway from OHC (G1) to MHD (G2). This latter pathway was added in model D but did not substantially improve model fit. While this is an interesting finding in itself, we chose to focus on the results from model D since it provides a more comprehensive picture of the issue. In order to have a more complete understanding of the coefficients presented in Table 2, we show the crude estimate for each pathway separately (full sample and stratified by sex (G2)) in the supplementary material (SM 2).

**Table 1:** Fit statistics contrasting models A, B, C, and D.

	Model A	Model B	Model C	Model D
	BIC/AIC	BIC/AIC	BIC/AIC	BIC/AIC
Full sample	17639.72 /17532.82	17646.13 /17531.59	**17544.53** /17429.99	17550.94 /**17428.76**
Grouped by sex in G2	17723.46 /17540.2	17739.44 /17540.91	**17636.21 /17437.68**	17652.19 /17438.38

AIC = Akaike information criterion; BIC=Bayesian information criterion.

**Table 2:** Generalised Structural Equation Modelling (GSEM) model investigating the associations between out-of-home care (OHC) and mental health disorders (MHD). Full sample and stratified by sex (G2) (models C and D).

	Full sample (N = 15,305)		G2 Males (N = 7913)		G2 Females (N = 7392)	
OHC (G1)→	Model C	Model D	Model C	Model D	Model C	Model D
	OR (95% CI)	OR (95% CI)	OR (95% CI)	OR (95% CI)	OR (95% CI)	OR (95% CI)
→MHD (G1)	2.39 (1.77–3.22)	2.39 (1.77–3.22)	3.02 (2.04–4.46)	3.02 (2.04–4.46)	1.79 (1.12–2.87)	1.79 (1.12–2.87)
→OHC (G2)	3.66 (2.75–4.88)	3.66 (2.75–4.87)	3.22 (2.14–4.85)	3.22 (2.14–4.86)	4.24 (2.84–6.32)	4.24 (2.84–6.33)
→MHD (G2)	–	1.37 (0.98–1.90) ^n.s.^	–	1.42 (0.89–2.26) ^n.s.^	–	1.32 (0.83–2.11) ^n.s.^
MHD (G1)→						
	OR (95% CI)	OR (95% CI)	OR (95% CI)	OR (95% CI)	OR (95% CI)	OR (95% CI)
→MHD (G2)	2.16 (1.40–3.35)	2.11 (1.36–3.27)	2.46 (1.36–4.44)	2.36 (1.30–4.28)	1.92 (1.00–3.67)	1.90 (0.99–3.63) ^n.s.^
→OHC (G2)	8.14 (5.83–11.36)	8.14 (5.83–11.36)	6.87 (4.25–11.09)	6.87 (4.25–11.10)	9.85 (6.19–15.68)	9.85 (6.19–15.69)
OHC (G2)→						
	OR (95% CI)	OR (95% CI)	OR (95% CI)	OR (95% CI)	OR (95% CI)	OR (95% CI)
→MHD (G2)	3.79 (2.54–5.64)	3.60 (2.40–5.38)	5.08 (3.03–8.54)	4.84 (2.86–8.18)	2.69 (1.44–5.05)	2.56 (1.36–4.83)

^n.s.^: not statistically significant.

Presented estimates are drawn from models C and D (Fig. 1).

Wald tests did not show statistical differences between G2 males and G2 females in any of the pathways.

All models were adjusted for occupational class (G1), sex (G1) and sex (G2 - Full sample).

As demonstrated in Table 2, there was an intergenerational transmission of OHC to both G2 males and G2 females; this transmission appeared to be slightly stronger among females (G2 males: OR 3.22, 95% CI 2.14–4.86; G2 females: OR 4.24, 95% CI 2.84–6.33), although there were no statistically significant differences (CIs overlapped). While the results suggested transmission of MHD from G1 to G2 in the full sample, this association was only statistically significant among G2 males in the sex-stratified analysis (G2 males: OR 2.36, 95%, CI 1.30–4.28; G2 females: OR 1.90, 95% CI 0.99–3.63). There was an association between OHC and MHD within both generations; in G2, this association was stronger among males (males: OR 4.84, 95% CI 2.86–8.18; G2 females: OR 2.56, 95% CI 1.36–4.83), but no statistically significant differences were evident based on the overlapping CIs. Moreover, the results clearly show that MHD in G1 is associated with OHC in G2, with significantly larger estimates for G2 females (G2 males: OR 6.87, 95% CI 4.25–11.10; G2 females: OR 9.85, 95% CI 6.19–15.69- CIs did not overlap). On the other hand, there was no significant association between OHC in G1 and MHD in G2 (G2 males: OR 1.42, 95% CI 0.89–2.26; G2 females: OR 1.32, 95% CI 1.32, 95% CI 0.83–2.11). We nonetheless compared the estimates from the intergenerational associations in Model D with the crude estimates of each pathway (SM 2). The latter showed a significant association between OHC in G1 and MHD in G2, which might lead to the conclusion that there is an indirect influence that is explained by the indirect pathway through MHD in G1 and/or OHC in G2.

## 4 Discussion

The main purpose of this study was to investigate associations between OHC and MHD across two generations, stratified by sex. First of all, there was an intergenerational transmission of OHC and MHD respectively, although the latter was primarily evident among G2 males. The lack of sex differences in the transmission of OHC is in line with an earlier Danish study (Mertz and Andersen, 2017), whereas the more evident intergenerational transmission of MHD among males is contrary to what other studies have found (Andreas et al., 2018; Gonçalves et al., 2016). This discrepancy could be explained by e.g. differential follow-up times and data sources. Furthermore, the results of the current study confirmed associations between OHC and MHD within each generation. For G2, this was particularly the case among males, which contrasts with the majority of studies that reveal that rates of MHD are particularly high in females with experiences of OHC compared to reference populations without such experiences (Hambrick et al., 2016; K ää ri ä l ä and Hiilamo, 2017; Vinnerljung and Hjern, 2018; Gypen et al., 2017; Lee and Holmes, 2021; Conn et al., 2015).

Concerning the intergenerational associations between OHC and MHD, children – and especially daughters – to parents with mental health disorders were more likely to experience placement in OHC. Existing evidence has documented a strong relationship between parental mental illness and involvement with child protective services (Hammond et al., 2017; Park et al., 2006; O’ Donnell et al., 2015; Simoila et al., 2019; Kohl et al., 2011). Corroborating our findings, an Australian study by O’Donnell and colleagues (O’ Donnell et al., 2015) found that maternal history of MHD was associated with a more than doubled risk of involvement with child protective services; girls appear to have a higher risk than boys of being placed, although the mechanisms have not been sufficiently illuminated. Another study from the US using administrative data found that maltreatment reports and foster placements were more likely for children of mothers with mental illness than for those born to mothers without mental illness, regardless of child sex (Kohl et al., 2011).

The opposite direction of the intergenerational associations was not as evident: we could not identify any direct statistical effect of OHC placements among parents on their children's MHD in adulthood. Rather, there was some indication of indirect effects, operating via parents’ MHD and/or OHC among the children. Thus, the lack of a direct effect does not mean that the adversities faced by an antecedent generation do not influence the mental health of their descendants. By systematically reviewing the intergenerational effect of maternal childhood maltreatment on offspring's vulnerability to MHD, previous studies have found a small but significant effect in this association, which was further attenuated by maternal depression (Su et al., 2020).

The result might also be discussed in conjuncture with the ecobiodevelopmental theory as a way to raise hypotheses that help to explain the impact of being placed into care in one generation and the mental health vulnerability of their children: (a) the transmission effect could be inherited through epigenetic alterations in genes (more prone to occur during the gestation, early and reproductive periods) which moderates the effects of environmental factors on the risk of childhood expression of MHD (Park et al., 2019; Guintivano and Kaminsky, 2016); b) prenatal childhood adversities may lead to disruptions in stress regulation abilities and change the brain structures and functioning. It is worth noting that the stress-response system (hypothalamic-pituitary-adrenal (HPA) axis) activated when facing strain situations varies between individuals and sexes: females tend to have a stronger HPA axis response to social stress in childhood, while young males have higher HPA axis responses to psychological stress in adulthood (Heim et al., 2008); c) children of parents with history adversities might be more likely to experience maltreatment, and consequent OHC placement, via mechanisms such as inadequate care, dysfunctional parents–child relationships, and unstable housing which can culminate in vulnerability in offspring to MHD (Plant et al., 2018; Su et al., 2020). Moreover, these potential mechanisms might support our findings which suggest that MHD (G1) and OHC (G2) are indirect pathways that may be explaining the lack of direct association between OHC (G1) and MHD (G2) in the cross-lagged model, at least for sons. It also corroborates the findings from our previous study (Straatmann et al., 2021) which showed a fundamental role of parental MHD in the intergenerational transmission of OHC.

### 4.1 Strengths and limitations

To the best of our knowledge, this is the first study to investigate the associations between OHC and MHD both within and across generations, including the explicit assessment of whether parental OHC experiences translate into MHD among their adult children. We used comprehensive prospective data with low attrition in which the individuals’ (and their children's) participation in the study did not depend on having OHC experience and/or poor mental health. Nonetheless, our birth cohort includes a large number of individuals with experience of OHC. Another advantage is that we did not rely on retrospective self-reports or parental reports about experiences of OHC. It is worth noting that most studies on this topic analyse maternal circumstances concerning their children, whereas our study instead used information on both mothers and fathers. Despite that, more research is needed on the impact of parent-child sex (a)symmetry and its effects on the parental adverse experiences and offspring's mental health relationship (Su et al., 2020).

Some limitations should be addressed. First, caution must be taken with the generalizability of results and comparability between the measurements of OHC placements across generations. There was a lack of information about children who were sent to foster families residing outside the Stockholm area and those who were born outside Stockholm and exposed to child welfare measures before they moved to Stockholm. Our data also lack information on both parents; in other words, the G2 individual has information about the G1 mother or the G1 father (whichever parent was included in the SBC Multigen), but not both. The reduction of the sample size due to the restrictions in birth years, age of placements and age of MHD occurrence in G1 and G2 might be a limitation.

While potential genetic aspects are important in the intergenerational transmission of mental and behavioural disorders, the available data does not allow us to explore these aspects further. Nevertheless, we attempted to be cautious about genetic explanations by excluding MHD with an early onset and high heritability. Although information about the mental health of both generations is from the same source (Patient Registers; inpatient care), differences in diagnosis and routine around MHD must be taken into consideration when discussing these findings. Last but not least, cross-lagged panel models inherently involve claims about causality. While we did our best to preserve a reasonable temporal ordering in the data and, for example, excluded cases where MHD (G1) occurred after the OHC placement (G2), caution with a causal interpretation of the results is recommended.

### 4.2 Implications

While parents’ MHD seems to directly influence children's OHC placement, particularly among their daughters, this study did not reveal any direct intergenerational path going from parental OHC to MHD in the next generation of adult children. For the latter, the indirect pathways nevertheless seem to play an important part, emphasising the role of parents’ mental health and the children's own placement experiences. The reasons underlying the greater vulnerability of girls to experience OHC given their parents' mental health must be further explored. Girls in OHC are a high-risk group (Dowdell et al., 2009); it might be the case that those girls were more exposed to maltreatment or neglect due to their parents MHD leading to a higher risk of placement compared to boys.

Furthermore, mental health should receive special attention, particularly among those with a prior history of OHC. Despite the empirical support and clinical importance of MHD in children involved in OHC, it is still surprising that only a few nations have regulations or standardised practices for the assessment and monitoring of children's mental health while entering into care or in societal care (Vinnerljung and Hjern, 2018). Health professionals and social workers should be able to develop effective and timely interventions to support these individuals struggling with mental health issues and to reduce the risk of MHD; it might help to break cycles of disadvantages and promote positive outcomes for future generations. In Sweden, although there is a regulation in place to ensure health examinations for children entering into care, it has not been properly followed by local authorities. In order to ensure such services to children, regions and municipalities need to cooperate. Therefore, agreements between regions (e.g. health care services) and municipalities (i.e. social services) have been put forward to ensure cooperation between these sectors, but many efforts are still needed to guarantee complete health check-ups and follow-ups for these children (Kazemi, 2021).

## 5 Conclusion

We conclude that OHC and MHD seem to be processes intertwined both within and across generations, with some variation according to sex. Although there did not seem to be any direct influences of OHC in one generation on MHD in the next generation, there was some indication of indirect paths going via parental MHD and child OHC. This study highlights the importance of offering appropriate mental health support not only to children but also to parents with MHD, particularly those with a history of OHC placement. It might either impact the household environment where children grow up (i.e. preventing children's removal from biological parents) as well as the likelihood of eventually successful reunifications. Future research should provide further knowledge that might be used by both institutions (i.e. mental health services and child welfare services) to strengthen collaboration between these actors and appropriate services for vulnerable parents and children.

## Ethical statement

The Regional Ethical Review Board in Stockholm approved the creation of RELINK53 as well as the probability matching to the SMS that resulted in SBC Multigen (no. 2017/34–31/5; 2017/684–32). Statistics Sweden, along with the other governmental agencies that were asked to provide data, approved of the new matching, extensions and data extractions.

The funding source has not been involved in the study design; analysis and interpretation of data; in the writing of the articles; and in the decision to submit it for publication.

## CRediT authorship contribution statement

**Viviane S. Straatmann:** Conceptualization, Methodology, Formal analysis, Writing – original draft, Visualization, Project administration, Funding acquisition. **Josephine Jackisch:** Conceptualization, Writing – review & editing, Funding acquisition. **Lars Brännström:** Conceptualization, Writing – review & editing, Funding acquisition. **Ylva B. Almquist:** Conceptualization, Methodology, Writing – review & editing, Supervision, Project administration, Funding acquisition.

## Declaration of competing interest

None.
