# Supplementary material for: Associations between out-of-home care and mental health disorders within and across generations in a Swedish birth cohort
Source: SSM Popul Health. 2022 May 8;18:101115. doi: 10.1016/j.ssmph.2022.101115 (PMC9118912; doi:10.1016/j.ssmph.2022.101115)
Supplement: Multimedia component 1 [file mmc1.docx]

**Supplementary material**

SM 1. Characteristics of the study sample by sex- tabulation of Figure 2.

|  | Generation 1 | | | Generation 2 | | |
| --- | --- | --- | --- | --- | --- | --- |
|  | Full sample  n= 9,033 | Males  n= 4,072 | Females  n= 4,961 | Full sample  n= 15,305 | Sons  n= 7,913 | Daughters  n= 7,392 |
|  | n (%) | n (%) | n (%) | n (%) | n (%) | n (%) |
| **Out-of-home care (OHC)** |  |  |  |  |  |  |
| No | 8,430 (93.32) | 3,788 (93.03) | 4,642 (93.57) | 15,004 (98.03) | 7,758 (98.04) | 7,246 (98.02) |
| Yes | 603 (6.68) | 284 (6.97) | 319 (6.43) | 301 (1.97) | 155 (1.96) | 146 (1.98) |
| **Mental health disorders (MHD)** |  |  |  |  |  |  |
| No | 8,826 (97.71) | 4,004 (98.33) | 4,822 (97.20) | 14,888 (97.28) | 7,714 (97.49) | 7,174 (97.5) |
| Yes | 207 (2.29) | 68 (1.67) | 139 (2.80) * | 417 (2.72) | 199 (2.51) | 218 (2.95) |
| **Childhood occupational class** |  |  |  |  |  |  |
| Middle-upper class | 4,293 (47.53) | 1,897 (46.59) | 2,396 (48.30) | - | - | - |
| Working class | 4,740 (52.47) | 2,175 (53.41) | 2,565 (51.70) | - | - | - |

*Chi-square test; there was only a statistically significant difference (p<0.05) among males and females regarding MHD in G1.

SM 2. Estimates focused on single pathways of out-of-home care (OHC) and mental health problems (MHP) across generations. Full sample and stratified by sex (G2).

|  | **Full sample**  **(N=15,305)** | **G2 Male**  **(N=7,913)** | **G2 Female**  **(N=7,392)** |
| --- | --- | --- | --- |
|  | OR (95% CI) | OR (95% CI) | OR (95% CI) |
| OHC (G1)🡪MHP (G1) | 2.39 (1.77-3.22) | 3.02 (2.04-4.46) | 1.79 (1.12-2.87) |
| OHC (G1)🡪OHC (G2) | 4.09 (3.06-5.41) | 3.72 (2.50-5.55) | 4.50 (3.05-6.66) |
| OHC (G1)🡪MHP (G2) | 1.59 (1.15-2.19) | 1.73 (1.10-2.72) | 1.46 (0.92-2.31) |
| MHP (G1)🡪MHP (G2) | 2.77 (1.82-4.22) | 3.28 (1.87-5.76) | 2.31 (1.24-4.33) |
| MHP (G1)🡪OHC (G2) | 9.19 (6.63-12.73) | 8.02 (5.02-12.82) | 10.54 (6.69-16.60) |
| OHC (G2)🡪MHP (G2) | 4.35 (2.96-6.39) | 5.88 (3.56-9.72) | 3.06 (1.67-5.61) |

Estimates generated through GSEMs adjusted for childhood occupational class (G1) and sex (G1).
